# Consideration of Oral Health and Periodontal Diseases During Pregnancy: Knowledge and Behaviour Among French Pregnant Women

**DOI:** 10.3290/j.ohpd.b875513

**Published:** 2021-01-26

**Authors:** Catherine Petit, Juliette Benezech, Jean-Luc Davideau, Virginie Hamann, Nicolas Tuzin, Olivier Huck

**Affiliations:** a Associate Professor, Université de Strasbourg, Faculté de Chirurgie Dentaire, Periodontology, Strasbourg, France; Pole de médecine et chirurgie bucco-dentaire, parodontologie, Hôpitaux Universitaires de Strasbourg, Strasbourg, France. Analysed the clinical data; wrote the draft.; b Student Midwife, Ecole des Sages-femmes, Hôpitaux Universitaires de Strasbourg, Strasbourg, France. Collected the data; edited and commented on the manuscript.; c Professor, Université de Strasbourg, Faculté de Chirurgie Dentaire, Periodontology, Strasbourg, France; Pole de médecine et chirurgie bucco-dentaire, parodontologie, Hôpitaux Universitaires de Strasbourg, Strasbourg, France. Conceived the study, analysed the clinical data; wrote the draft.; d Midwife, Ecole des Sages-femmes, Hôpitaux Universitaires de Strasbourg, Strasbourg, France. Collected the data; edited and commented on the manuscript.; e Engineer, Groupe de Méthodes en Recherche Clinique, Hôpitaux Universitaires de Strasbourg, Strasbourg, France. Analysed the clinical data; edited and commented on the manuscript.; f Professor, Université de Strasbourg, Faculté de Chirurgie Dentaire, Periodontology, Strasbourg, France; Pole de médecine et chirurgie bucco-dentaire, parodontologie, Hôpitaux Universitaires de Strasbourg, Strasbourg, France. Conceived the study, analysed the clinical data; wrote the draft.

**Keywords:** oral health, questionnaire, prevention, risk factor

## Abstract

**Purpose:**

Several studies demonstrated compromised oral health and periodontal diseases as risk factors for adverse pregnancy outcomes. However, consideration of oral health by pregnant women remains elusive. The aim of this study was to evaluate knowledge and behaviour of French pregnant women towards relationship between oral conditions and pregnancy outcomes and to evaluate influencing factors.

**Materials and Methods:**

A self-reported questionnaire was given to women between 1 and 3 days after delivery in three specialised clinics in France. The questionnaire aimed to evaluate demographic characteristics, self-perceived oral health, type of pregnancy follow-up and knowledge regarding oral conditions during pregnancy and risk of adverse pregnancy outcomes. A multivariate analysis was performed to evaluate correlation between knowledge and behaviour.

**Results:**

The questionnaire was completed by 212 women. Among them, 92% considered prevention of oral diseases during pregnancy important. Despite knowledge of potential negative influence of periodontal diseases on pregnancy outcomes, only 47% of pregnant women received dental diagnosis or treatment during pregnancy. Only 18% of the women discussed oral health consideration during pregnancy with health professional in charge of pregnancy follow-up. Interestingly, absence of dental consultation during pregnancy was associated with low rate of dental consultation prior to pregnancy (p < 0.01).

**Conclusions:**

Pregnant women were aware of the association between oral health and pregnancy and of need of prevention. However, consideration of importance of oral health was not adequate to the rate of dental consultation and seems to be influenced by individual dental follow-up habits prior to pregnancy.

**Clinical Relevance:**

Dental evaluation should be considered systematically during pregnancy follow-up.

Several studies highlighted the bidirectional link between oral conditions and pregnancy as pregnant woman are more prone to have compromised oral health.^26^ Indeed, a higher risk of caries and periodontal diseases as well as their local morbidity are observed.^28^ Pregnancy is clinically associated to an increase of the prevalence and/or the severity of the gingivitis characterised by gingival bleeding and swelling and of the prevalence and/or the severity of periodontitis, characterised by the increase of periodontal probing depth, clinical attachment and bone losses.^4,26,32^ Caries and periodontal diseases are defined as infectious diseases due to oral biofilms: supragingival biofilm for caries, supragingival and subgingival biofilms for periodontal diseases.^37^ During pregnancy, the quantity and virulence of the oral microbiome are modified as more viable counts are observed, such as detection of well-described periodontal pathogens *Porphyromonas gingivalis* and *Aggregatibacter actinomycetemcomitans*.^35^

Moreover, the systemic influence of periodontal diseases on adverse pregnancy outcomes, including miscarriage, pre-eclampsia, preterm birth and low birth weight, has also been shown^14,34,36^ even if it remains still under investigation.^19^ Adverse pregnancy outcomes, including preterm birth, are considered the leading cause of neonatal mortality^30^ and around 6% of births in France occurred before 37 weeks.^11^ The main hypotheses linking these two conditions are the bacterial spreading and the sustained chronic cytokines release from periodontal lesions to foeto-maternal unit but also shared risk factors such as smoking or obesity.^26^

The course of pregnancy is a particular experience for women and its management comprises prevention and control of identified risk factors, such as smoking or obesity, to reduce risk of adverse pregnancy outcomes^12,20^ but also to prevent all events that may affect quality of life. Accordingly, smoking cessation strategies are of importance in the global management well-known by pregnant women.^29^ Recommendations regarding oral health are also part of the pregnant women follow-up,^3,24^ however this topic is not systematically discuss with pregnancy professionals (obstetricians, gynaecologists, midwives, nurses or physicians) despite awareness of its importance.^2,13,21,22,38,43,44^ Unfortunately, most of available data showed that pregnant women have a poor knowledge and misconception regarding oral health and dental treatment during pregnancy.^41,42^ This lack of knowledge may be due to insufficient information received during the pregnancy follow-up as oral health is not often discuss with patients. This results in disparities regarding dental care use among pregnant women as observed in USA (70%)^44^ and in France (56%).^42^ Such consultation rate during pregnancy could be considered as relatively low, despite a preventive program including free dental consultation as established in France. Interestingly, several influencing factors have been identified explaining this lack of dental treatment such as socioeconomic factors or women’s lack of receiving dental treatment before pregnancy.

The aim of this study was to evaluate periodontal knowledge and behaviour related to the oral health of pregnant women and to determine influencing factors during pregnancy follow-up in a French population.

## Materials and Methods

### Study Design and Population

This study was performed using a self-administered questionnaire constituted 38 questions assessing demographical data, pregnancy follow-up, oral hygiene habits and oral and periodontal diseases-related knowledge. The questionnaire was pilot-tested to validate and to ensure full comprehension of the questions. The protocol of this study was approved by Institutional Review Board (ref: 2017-86). Questionnaires were randomly submitted in three specialised obstetrical hospitals in France, during a 3-month time period, to women between 1 and 3 days after delivery in France. Participants entered the study voluntarily following an explanation of its objectives. Questionnaire, in French, included multiple-choice, yes/no and Likert scale questions. Around 15 min were necessary to answer all questions.

### Statistical Analysis

Data were analysed using a personal computer-based software. A descriptive analysis first presented the characteristics of the patients. The quantitative variables were described using the mean and standard deviation. The qualitative variables were presented in terms of numbers and percentages. In a second step, a univariate analysis was carried out between the different questions. The qualitative variables were compared using the Chi-square test if the conditions were met, otherwise the exact Fisher test. The quantitative variables were compared using one-way analysis of variance (ANOVA) if the conditions were met, otherwise the Kruskal-Wallis test. The Gaussian distributions of the quantitative variables were evaluated graphically and using the Shapiro-Wilk test. Finally, multivariate analyses were performed using logistic regression when the dependent variable had two groups and multinomial regression when the dependent variable had at least three groups. For each regression, the odds ratio (OR) with its 95% confidence interval was presented. A p value < 0.05 was considered statistically significant. The analyses were carried out using software R version 3.5.0.

## Results

### Demographic Data and Obstetrical Follow-Up

Demographic and pregnancy follow-up characteristics are presented in Tables 1 and 2. A total of 212 women were accepted to enter the study. Seventy-five per cent (75%) of the population were between 20 and 35 years old and 24% were over 35 years. Only 2 women were under 20 years at delivery. Delivery occurred after 37 weeks for 90% of the patients while 10% occurred between 32 and 36 weeks. Most of the participants were considered non-obese as only 68 (32%) had a body mass index (BMI) over 25 and only 3 women were considered as heavy smokers (> 10 cig/day). Eighty-three (39%) participants were having their first pregnancy, 60 (28%) their second and 69 more than 2 (33%).

**Table 1:** Demographic characteristics

Demographic characteristics	Study’s population
Age (years) (n; (%) <20 20–35 >35	2 (1) 159 (75) 51 (24)
Weight before pregnancy (kg) (mean; (SD))	65.4 (13.2)
BMI (mean; (SD))	23.9 (4.5)
Smoking (n; (%)) Yes No	29 (14) 183 (86)
Profession (n; (%)) Higher managerial and professional occupations Intermediate occupation Routine occupation Non-working Student	37 (17.5) 42 (20) 82 (39) 49 (23) 1 (05)

**Table 2:** Characteristics of pregnancy

Pregnancy	Study’s population
Number of pregnancy (n; (%)) 1 2 3 >3	83 (39) 60 (28) 32 (15) 37 (18)
Number of children (n; (%)) 1 2 3 >3	100 (47) 69 (33) 24 (11) 19 (9)
Pregnancy follow-up by: (n; (%)) Physician Midwife Gynaecologist/obstetrician	10 (4.7) 68 (32) 172 (80)
Time of delivery (n; (%)) >37 weeks between 32 and 36 weeks <32 weeks	188 (90) 20 (10) 0 (0)

For 68.8% of the women, pregnancy follow-up was performed by an obstetrician/gynaecologist only, 27.2% by a midwife only and less than 4% was exclusively followed by a physician. No influence of age, weight and smoking status has been observed on the type of pregnancy follow-up or time of delivery (p >0.05).

### Periodontal Diseases Knowledge and Awareness

For 92% of the women, prevention of oral diseases during pregnancy is considered important. However, during pregnancy, information related to oral health has been given by pregnancy professionals to only 18.2% of the sample population. Regarding potential influence of low oral hygiene/periodontal diseases on adverse pregnancy outcomes, 35.6% of the population was aware of an increased risk and cited bacterial spreading from oral cavity to foeto-placenta unit as a biological explanation. Interestingly, media such as TV, magazines or newspapers were the source of knowledge for 16% of the sample (Table 3).

**Table 3:** Pregnant woman’s knowledge

Oral disease/pregnancy interactions awareness	Study’s population (n; [%])
Oral health discussed with professional in charge of pregnancy follow-up Yes No	35 (18.2) 157 (81.8)
Are you aware of the feasibility of dental consultation during pregnancy? Yes No	165 (80.9) 39 (19.1)
Is the prevention and treatment of oral diseases important during pregnancy? Yes No	188 (92) 16 (8)
Are you aware of putative influence of oral hygiene, periodontal disease and adverse pregnancy outcomes? Yes No	72 (35.6) 130 (64.4)
Which mechanisms could explain this link? Bacterial spreading Dental pain induces uterine contraction Release of inflammation/infection by-products induced inflammation at distance Anxiety that increase uterine contractions Fatigue associated to oral disease impeach delivery Don’t know	74 (44.6) 25 (15.1) 21 (12.6) 6 (3.8) 1 (0.6) 80 (43.2)
Do you think that dental follow-up/treatment during pregnancy is of importance? (n; (%)) Yes No	188 (92.2) 16 (7.8)

### Oral Health Status During Pregnancy

Most of the participants were felt to have a good oral health (score >7 for 63%) (Table 4). This score is commensurate with oral hygiene habits, as 74% declared to brush their teeth at least two times per day for at least 2 min (62%) and with fluoride toothpaste (51%). Only 31% of the pregnant women used interdental brushes or floss. Around 70% consult their dentist at least once per year with approximatively 55% of them for routine check-up and treatments (scaling and caries treatment). Only 7 patients were followed for periodontal diseases prior to pregnancy. However, during pregnancy, 99 pregnant women (47%) have been seen by their dentist, mostly for check-up. Interestingly, the reasons for not being willing to be seen by a dentist were the absence of dental symptoms (42.7%), the lack of time (25%) or the lack of information regarding feasibility of dental treatment during pregnancy (14%). Most of the consultations or dental treatments have been performed during the second trimester. Multivariate analysis demonstrated that age and self-perceived oral health status were associated to the use of fluoride toothpaste, use of interproximal devices and frequency of dental check-up (Table 5).

**Table 4:** Attitude and behaviour during pregnancy

Oral health related characteristics	Study’s population (n; [%])
Self-perception of oral health (1 = very poor; 10; very good) Mean (SD) <4 (poor) 4 to 7 (average) >7 (good)	5 (2.4) 72 (34.6) 131 (63)
Toothbrushing (n/day) >2/d 1/d <1/d	156 (74) 52 (24) 3 (1.4)
Dental consultation (n/year) >2/yr 1/yr <1/yr Never	35 (17) 112 (53) 54 (25.5) 10 (4.5)
Presence of periodontal disease before pregnancy (n; (%)) Yes No	7 (3.3) 204 (96.7)
Adverse oral condition during pregnancy Dental erosion Caries Periodontitis Gingivitis Epulis Pain Abscess Other	6 (5.6) 39 (36.5) 19 (17.8) 53 (49.5) 1 (0.9) 7 (6.5) 5 (4.7) 3 (2.8)
Dental consultation during pregnancy Yes No	99 (47) 112 (53)
Dental treatment during pregnancy Yes No	53 (25) 158 (75)

**Table 5:** Analysis of factors associated with oral hygiene habits of the participants during pregnancy (* p <0.05 after multivariate analysis). N corresponds to number of respondents related to demographic characteristics

Question	Demographic and periodontal characteristics	Respondents (N)	Answer		p value
			Yes n(%)	No n(%)	
Are you using fluoride toothpaste?	**Age**				
	<25 years old	2	2 (100)	0 (0)	0.004* (OR = 0.39)
	25–35 years old	156	70 (44.9)	86 (55.1)	
	>35 years old	50	34 (68)	16 (32)	
	**Oral health status note**				
	From 1 = very bad to 10 = excellent (mean= 7.6)	205	105 (51.2)	100 (48.8)	0.44
Are you using dental floss or interdental brushes?	**Age**				
	<25 years old	2	0 (0)	2 (100)	0.02* (OR = 0.45)
	25–35 years old	159	43 (27)	116 (73)	
	>35 years old	51	23 (45.1)	28 (54.9)	
	**Oral health status note**				
	From 1 = very bad to 10 = excellent (Mean 7.6)	208	64 (30.7)	144 (69.3)	0.26

Question	Demographic and periodontal characteristics	Respondents (N)	Answer			p value
			Less than 6 months	Between 6 and 12 months	More than 1 year	
The reasons why you consult a dentist is for a regular check-up	**Oral health status score**					
	From 1 = very bad to 10 = excellent	208	78 (37.5)	125 (60.1)	5 (2.4)	0.0002* (OR = 1.3786)
The reasons why you visit a dentist is related to toothache/ pain	**Oral health status score**					
	From 1 = very bad to 10 = excellent	208	63 (30.3)	140 (67.3)	5 (2.4)	**0.002**
The reasons why you visit a dentist is related to bleeding while brushing	**Oral health status score**					
	From 1 = very bad to 10 = excellent	208	173 (83.2)	30 (14.4)	5 (2.4)	0.57
The reasons why you visit a dentist is related to the free consultation proposed during pregnancy	**Oral health status score**					
	From 1 = very bad to 10 = excellent	208	165 (79.3)	38 (18.3)	5 (2.4)	**0.04**

Question	Demographic and periodontal characteristics	Respondents (N)	Answer				p value
			2 to 3 times/ year	Once a year	Less than once a year	Never	
How often do you consult a dentist?	**Age**						
	<25 years old	2	0 (0)	0 (0)	1 (50)	1 (50)	0.11
	25–35 years old	158	7 (4.4)	43 (27.2)	85 (53.8)	23 (14.6)	
	>35 years old	51	2 (3.9)	10 (19.6)	27 (52.9)	12 (23.5)	

### Influencing Factors Associated with Knowledge Towards Oral Health During Pregnancy

To determine which factor influences oral health related knowledge of pregnant women, a multivariate analysis has been conducted including all potential influencing factors identified through a univariate analysis with a p value <0.2 (Table 6). Women aware of a possible dental consultation during pregnancy were more prone to have a high self-perceived oral health status (OR = 1.23), regularly consult their dentist, and have been followed by an obstetrician/gynaecologist (OR = 2.33). Knowledge related to the influence of oral hygiene and oral diseases on pregnancy outcomes was more important in women having attended dental consultation recently (<1 year), having a high self-perceived oral health status, having been followed by an obstetrician/gynaecologist and having several children. Interestingly, women considering prevention and management of dental diseases during pregnancy of importance have been seen during pregnancy by their dentist (<1 year) (OR = 0.11).

**Table 6:** Analysis of factors associated with patient’s knowledge during pregnancy (* p < 0.05 after multivariate analysis). N corresponds to number of respondents related to demographic characteristics

Question	Demographic and periodontal characteristics	Respondents (N)	Answer		p value
			Yes n (%)	No n (%)	
Patient knows that a dental consultation is possible during pregnancy	**Age**				
	<25 years old	2	0 (0)	2 (100)	**0.02**
	25–35 years old	154	123 (79.9)	31 (20.1)	
	>35 years old	48	42 (87.5)	6 (12.5)	
	**Oral health status note**				
	From 1 = very bad to 10 = excellent	201	163 (81.1)	38 (18.9)	0.048* (OR = 1.23)
	**Patients who never consult dentists**				
	Yes	10	2 (20)	8 (80)	0.0003* (OR = 0.03)
	No	194	163 (84.1)	31 (15.9)	
	**When was your last visit to the dentist**				
	Less than 1 year	149	127 (85.2)	22 (14.7)	**0.03**
	More than 1 year	55	38 (69.1)	17 (30.9)	
	**Pregnancy follow-up by a midwife**				
	Yes	66	51 (77.3)	15 (22.7)	**0.45**
	No	138	114 (82.6)	24 (17.4)	
	**Pregnancy monitoring by obstetrician or gynaecologist**				
	Yes	165	138 (83.6)	27 (16.4)	0.041* (OR = 2.33)
	No	39	27 (69.2)	12 (30.7)	
Patient knows that there may be a link between oral hygiene or gum diseases and adverse pregnancy outcomes	**When was your last visit to the dentist**				
	Less than 1 year	147	63 (42.8)	84 (57.1)	0.0008* (OR = 0.24)
	More than 1 year	55	9 (16.4)	46 (83.6)	
	**Pregnancy follow-up by a midwife**				
	Yes	66	51 (77.3)	15 (22.7)	0.75
	No	138	48 (34.8)	90 (65.2)	
	**Pregnancy monitoring by obstetrician or gynaecologist**				
	Yes	165	59 (35.8)	106 (64.2)	1.00
	No	37	13 (35.1)	24 (64.9)	
	**Pregnancy follow-up by a physician**				
	Yes	10	3 (30)	7 (70)	100
	No	192	69 (35.9)	123 (64.1)	
	**Did you consult a dentist during your pregnancy?**				
	Yes, after having received the invitation from health insurance	47	23 (48.9)	24 (51.1)	**0.04**
	Yes, for another reason	52	20 (38.5)	32 (61.5)	
	No	103	29 (28.1)	74 (71.9)	
Known link between periodontal disease and pre-eclampsia (high blood pressure, oedema, protein in the urine resulting from placental insufficiency)	**Oral health status score**				
	From 1 = very bad to 10 = excellent	49	11 (22.5)	38 (77.5)	**0.02 (OR = 2.82)**
	**How many children do you have so far**				
	From 1 to 5	49	11 (22.45)	38 (77.55)	0.04* (OR = 0.20)
Patients do not know how periodontal diseases can be linked to adverse pregnancy outcomes	**When was your last visit to the dentist**				
	Less than one year	121	49 (40.5)	72 (59.5)	0.009* (OR = 2.80)
	More than 1 year	45	31 (68.9)	14 (31.1)	
	Yes, for another reason	46	8 (17.4)	38 (82.6)	
	No	93	3 (3.3)	90 (96.7)	
Patients do not know that a link exists between oral hygiene and adverse pregnancy outcomes?	**Oral health status score**				
	From 1 = very bad to 10 = excellent	182	90 (49.5)	92 (50.5)	0.018* (OR= 0.80)
	**When was your last visit to the dentist**				
	Less than one year	137	56 (40.8)	81 (59.2)	0.000* (OR= 6.31)
	More than 1 year	47	36 (76.6)	11 (23.4)	
	**Pregnancy follow-up by a midwife**				
	Yes	147	76 (51.7)	71 (48.3)	0.46
	No	37	16 (43.2)	21 (56.8)	
	**Pregnancy follow-up by a physician**				
	Yes	6	2 (33.3)	4 (66.7)	0.68
	No	178	90 (50.6)	88 (49.4)	
Does the patient think that the management or prevention of dental problems in pregnant women is important?	**When was your last visit to the dentist**				
	Less than 1 year	152	149 (98.02)	3 (1.98)	0.0001* (OR = 0.1198)
	More than 1 year	52	39 (75)	13 (25)	
	**Pregnancy follow-up by a midwife**				
	Yes	67	58 (86.6)	9 (13.4)	0.045* (OR = 0.347)
	No	137	130 (94.9)	7 (5.1)	
	**Pregnancy monitoring by obstetrician or gynaecologist**				
	Yes	67	58 (86.6)	9 (13.4)	0.09
	No	137	130 (94.9)	7 (5.1)	
	**Pregnancy follow-up by a physician**				
	Yes	10	9 (90)	1 (10)	0.57
	No	194	179 (92.3)	15 (7.7)	

### Influencing Factors Associated with Oral Health Consideration During Pregnancy

Dental management of pregnant women is of importance, however, as observed previously all pregnant women did not attend dental consultation during pregnancy. Indeed, women with high self-perceived oral health status (OR = 1.30) were more prone to consult their dentist, while those having already several children consulted less (OR = 0.67) (Table 7). Interestingly, same factors were correlated with the experience of caries during pregnancy (OR = 0.48 and 1.80, respectively). It has been observed that women were used to visiting their dentist at least once per year for regular check-ups, and attended more dental consultations during pregnancy (p <0.001). However, no influence of pregnancy professionals having followed the pregnancy was observed.

**Table 7:** Analysis of factors associated with oral health examination, dental and periodontal treatment during pregnancy (*p <0.05 after multivariate analysis). N corresponds to number of respondents related to demographic characteristics

Question	Demographic and periodontal characteristics	Respondents (N)	Answer			p value
			Yes, after having received the invitation from Health Insurance	Yes, for another reason	No	
Did you consult a dentist during your pregnancy?	**Oral health status score**					
	From 1 = very bad to 10 = excellent	207	47 (22.7)	51 (24.6)	109 (52.6)	0.02* (OR = 1.2977)
	**How many children do you have so far**					
	From 1 to 5	211	47 (22.3)	52 (24.6)	112 (53.1)	0.0491* (OR = 0.6683)
	**Pregnancy follow-up by a midwife**					
	Yes	68	14 (20.6)	20 (29.4)	34 (50)	0.70
	No	143	33 (23.1)	32 (22.4)	78 (54.5)	
	**Pregnancy follow-up by a physician**					
	Yes	10	3 (30)	3 (30)	4 (40)	0.65
	No	201	44 (21.9)	49 (24.4)	108 (53.7)	
Patients who had caries during pregnancy	**Age**					
	<25 years old	0	0		0	0.44
	25–35 years old	61	21 (34.4)		40 (65.6)	
	>35 years old	22	10 (45.5)		12 (54.5)	
	**Oral health status score**					
	From 1 = very bad to 10 = excellent (Mean 7.6)	82	31 (24.4)		51 (62.2)	0.0001* (OR = 0.48)
	**How many children do you have so far**					
	From 1 to 5	83	31 (37.35)		52 (62.65)	0.031* (OR = 1.8)
	No	78	73 (93.6)		5 (6.4)	
No dental consultation during pregnancy because patients think it is not possible	**Age**					
	<25 years old	2	2 (100)		0 (0)	**0.03**
	25–35 years old	81	15 (18.5)		66 (81.5)	
	>35 years old	23	3 (13)		20 (87)	
	**Pregnancy follow-up by a midwife**					
	Yes	33	7 (21.2)		26 (78.8)	0.79
	No	73	13 (17.8)		60 (82.2)	
	**Pregnancy follow-up by a physician**					
	Yes	4	0 (0)		4 (100)	1.00
	No	102	20 (19.6)		82 (80.4)	
	No	102	2 (2)		100 (98)	
	Yes, for another reason	52	12 (23.1)		40 (76.9)	
	No	93	6 (6.45)		87 (93.55)	

## Discussion

In this study, it was observed that pregnant French women consider oral conditions of importance during the course of pregnancy. However, only a reduced proportion of the surveyed population attended dental consultation for preventive examination or dental treatment. Analysis of the influencing factors showed that management of oral conditions is more important for women having regular follow-ups at their dentist before pregnancy. Therefore, a specific emphasis should be made on women with irregular dental follow-up before pregnancy.

Oral health, especially associated to the presence of periodontal diseases, has impact on quality of life as demonstrated in general population.^18^ In pregnant women, it has been demonstrated that periodontal health and oral health-related quality of life are poorer than in non-pregnant women in several populations.^23,33^ Some contributing factors have been identified such as tooth loss, need of prosthesis but also the presence of caries^29^ highlighting the need of diagnosis and treatment early during pregnancy.

In this study, only 47% of the women visited their dentist during pregnancy. This reduced proportion of patients is in accordance with other study performed in France (44%)^33^ but also with studies conducted in USA or Greece.^9,16,39^ However, this proportion could be considered low, especially as in France, a free dental consultation is systematically proposed by health insurance during the second trimester. This reduced number of patients attending dental consultation may be due to lack of prevention by professionals in charge of pregnancy follow-up. As observed in this survey, less than 20% of the pregnant women discussed oral health during gynaecological consultation. This low consideration by professionals is related to different factors. In a study conducted among French obstetricians/gynaecologists, it was observed that these professionals have knowledge of the inflammatory and infectious nature of periodontal diseases and also considered bidirectional relationship between pregnancy and periodontal diseases.^13^ However, despite this reliable knowledge, a discrepancy between knowledge and clinical behaviour was observed. Indeed, most of the practitioners did not include in their consultation oral health-related interview due to lack of time and information but also because they don’t consider it a priority (22%). This result was in accordance with other surveys conducted in France on obstetricians and midwives.^10,17^

Treatment of oral diseases, especially periodontal diseases, is of importance before or during pregnancy. First of all, their efficacy and safety in terms of dental benefits and improvement of oral health-related quality of life is well described.^6^ However, the effect of periodontal treatment on pregnancy outcomes remains still under investigation. Regarding periodontal diseases, data have been conflicting. Interventional studies performed during pregnancy showed that periodontal treatment was safe and improved periodontal parameters related outcomes. It also reduced systemic inflammatory markers such as C-reactive protein (CRP) or IL-6.^27^ However, its impact on incidence of adverse pregnancy outcomes such as low birth weight or preterm birth has not been fully demonstrated yet, even if successful periodontal treatment has been correlated to full-term birth.^15,27^ False beliefs or misconceptions regarding dental treatments during pregnancy are still present in the population. In our study, around 20% of the surveyed patients were not aware of the safety and feasibility of dental care as observed in another study.^9^ This misbelief is also observable within dentists, as in France a recent survey showed that only 59.8% of practitioners believed that dental anaesthesia is not contraindicated and most practitioners believe that the best time for care is the second trimester or after pregnancy.^31^ Most importantly, some have concluded that even when dentists are aware of the association between periodontal diseases and complications of pregnancy, it doesn’t directly affect their preventive or treatment strategies.^13^ In this study, we also observed that knowledge and behaviour of pregnant women towards dental health and preventive care is influenced by several factors such as their own self-perceived dental status or regular follow-up by a dentist. However, no influence of a type of pregnancy professional having followed the pregnant women was determined, illustrating the need for reinforcing their consideration for oral health in the management of pregnant women.

The main limitation of this study is the reduced numbers of women with either low self-reported dental health but also women having faced an adverse pregnancy outcome, such as preterm birth. Inclusion of such women may be of interest to better understand their awareness and consideration for oral health as a potential risk factor for pregnancy.

During pregnancy, women are prone to change their noxious habits, thus encouraging preventive strategies. Multifaceted approaches could be used by non-dental professionals with interesting results. These approaches may combine different pedagogical tools including short video presentations, informative leaflets with provided oral hygiene instrumentations (toothbrush, toothpaste) and personalised counselling.^1^ As an example, it was demonstrated that the use of a short video explaining the principles of oral hygiene is effective to increase patient knowledge, at least at short term, as observed in the general population.^40^ Such counselling could be brief but should be performed routinely to enhance patient’s self-directed change process^25^ especially regarding oral hygiene improvement. Short-term educational programs have already shown their benefit on pregnant women’s beliefs and behaviours,^5^ however, a specific emphasis should be made on women not compliant with regular dental follow-up before pregnancy. Accordingly, preconception care should be envisaged as it has demonstrated benefit for both mothers and their children, especially, for women with other risk factors such as diabetes or obesity.^7^ Indeed, as suggested by the American Public Health Association, an opportunistic discussion could be started if ‘One Key Question’ determining a potential project of pregnancy is routinely asked to women of reproductive age especially in the context of gynaecological check-up.^8^

## Conclusions

Despite awareness of the importance of prevention and treatment of oral diseases during pregnancy, all pregnant women did not benefit from dental check-up or treatment. Effective preventive strategies should be proposed to allow the early diagnosis of periodontitis and also treatment of affected patients to reduce the associated risk of adverse pregnancy outcomes and to improve oral health-related quality of life during pregnancy. Such strategies should inform pregnant women during the first consultation for pregnancy follow-up but also women that are contemplating pregnancy.
